# Body dissatisfaction, excessive exercise, and weight change strategies used by first-year undergraduate students: comparing health and physical education and other education students

**DOI:** 10.1186/s40337-016-0133-z

**Published:** 2017-04-03

**Authors:** Zali Yager, Tonia Gray, Christina Curry, Siân A. McLean

**Affiliations:** 1 College of Education, Victoria University, Melbourne, 8001 Australia; 2 School of Education, Western Sydney University, Sydney, 2751 Australia; 3 School of Psychology and Public Health, La Trobe University; and College of Education, Victoria University , Melbourne, 3086 Australia

**Keywords:** Body dissatisfaction, Excessive exercise, Exercise disorder, Health and physical education, Teacher, Male, Disordered eating, Anabolic steroids

## Abstract

**Background:**

Health and Physical Education (HPE) teachers are known to be under social-, personal-, and employment-related pressure to be and appear physically fit, and to use dangerous dieting and weight control practices. This is problematic due to the influence this may have on their own health and the potential to model these attitudes and behaviours to their future students.

**Methods:**

In this paper, we compare the body image, dieting, disordered eating, and exercise behaviours of first year, HPE, and non-HPE, teacher education students. Participants were 596 first-year university student pre-service teachers (*n* = 249 HPE and *n* = 347 non-HPE) from three universities in Australia who completed self-report questionnaires. Analysis of covariance and logistic regression analyses were used to determine differences in attitudes and behaviours between these two groups for males and females separately.

**Results:**

We found that male HPE participants had significantly higher levels of drive for muscularity and obligatory exercise, and were more likely to be classified as having an exercise disorder, dieting, and using steroids than non-HPE students were. Female HPE students were more likely to engage in self-reported excessive exercise, to have higher scores on the Obligatory Exercise Questionnaire, and be classified as having an exercise disorder.

**Conclusion:**

These findings are important as they confirm the presence of dieting and disordered eating attitudes and behaviours among all teacher education students, and highlight male HPE teachers as a potentially vulnerable group. These results may inform the implementation of intervention programs for teacher education students to ensure their personal wellbeing and professional capacity in promoting positive body image, nutrition, and physical activity among young people.

**Electronic supplementary material:**

The online version of this article (doi:10.1186/s40337-016-0133-z) contains supplementary material, which is available to authorized users.

## Plain English Summary

In this study, we surveyed university students who were studying to become Health and Physical Education [HPE] teachers, or other teachers, and compared these two groups in terms of how they think and feel about their body, how much they want to be thinner or more muscular, and what sort of behaviours they are engaging in. We found that male HPE students were more likely to want to be muscular and to be engaging in excessive exercise, dieting, and using anabolic steroids than other male teacher education students. Female HPE students were more likely to be engaging in excessive exercise than other female education students. These findings mean that we should carefully consider the attitudes and behaviours of teachers who are presenting information about food and exercise in schools.

## Background

Most countries around the world include compulsory or elective health and/or physical education classes for students aged 5 to 16 years. In health classes, teachers are responsible for leading students through curriculum about nutrition and healthy food and lifestyle choices, alcohol and other drug use, mental health and wellbeing, relationships and sexuality, self-esteem, personal hygiene, and safety. In physical education, classes usually focus on the development of fundamental motor skills, engagement in games and sports, dance, and outdoor adventure activities. In Australia, a national curriculum has recently been established that combines the two areas of Health and Physical Education [1]. However in the USA and UK, Health Education, and Personal, Social, Health and Economic [PSHE] education (respectively), are separate from physical education. In Australia, Health and Physical Education [HPE] teachers are therefore responsible for educating young people with regard to food, physical activity, and mental health- all of which are relevant to the development of eating disorders.

Three decades of research into the personal eating and exercise behaviours of trainee and practicing Health and Physical Education (HPE) teachers reveal a need for concern. Although male and female HPE teachers have been found to be considerably more active than the average adult, and to have an average body mass index (BMI) that is at the lower end of the normal range [2], it is possible that this could be related to unhealthy weight control practices. O’Dea and Abraham [3] found that among female pre-service home economics and physical education (PE) teachers, 29% reported using excessive exercise, 19% used starvation, 22% induced vomiting, 19% used laxatives, and 7% used smoking to control their weight. Problematic attitudes and behaviours were also found among the males, as 29% desired weight gain to “bulk up” their muscles and 6% reported smoking for weight control [3]. Our earlier work also found that pre-service HPE teachers had a significantly higher prevalence of dieting, disordered eating and exercise behaviours than a control group of university students who were not enrolled in degrees related to food or exercise [4]. In particular, 41% of PE males and 32% of PE females were classified as having an exercise disorder according to cut-off levels of obligatory exercise [5] compared with 15 and 14% respectively in the non-PE group [4]. A New Zealand study found that female pre-service HPE teachers scored significantly higher on measures of dieting and bulimia nervosa and had significantly lower global self-esteem than female psychology students [6]. Finally, 18% of male PE majors in studies in the United States of America (USA) were considered to have high levels of body dissatisfaction and drive for muscularity [7].

HPE teachers may be engaging in these disordered dieting and exercise behaviours as they are under increased social [8], personal [9, 6], and employment-related [10] pressure to be and appear physically fit. HPE teachers are presumed and expected to embody the lean, muscular, ideal body shape [2, 10, 11]. Explicit external standards may also be imposed on pre-service HPE teachers in addition to the personal standards and expectations they may place on themselves for their academic, aesthetic, sporting, and social achievements. For example, some universities in the USA require fitness testing and strict body composition cut-offs to be met before allowing Physical Education (PE) students to graduate [12]. In addition, another study in the USA found that PE teachers who were less academically qualified but appeared to be physically fit were deemed more employable than those who were got higher test scores but were 5–10 kg overweight [13, 14]. This increased attention to, or an emphasis on body weight and shape for professional competence (either explicit or implicit) may therefore increase the risk of developing eating disorders [15].

Finally, a preoccupation with food and exercise has been reported to lead individuals towards food-related degrees and professions [16, 17]. In their classic study, Kinzl and colleagues [17] reported 14% of dieticians they surveyed had chosen that career partly due to their own preoccupation with food and exercise. There may be a similar phenomenon among those who want to become HPE teachers. Qualitative research with Australian first-year pre-service HPE teachers found that those studying HPE often report that they do so because of their love of sport, exercise, nutrition, and wanting to help people “to become as fit and healthy as I am” [18].

The body image, eating, and exercise behaviours of this specific group of university students are important because of the impact on the health and wellbeing of these individuals themselves. However there is also the potential for the attitudes and behaviours of these professionals to impact the health and wellbeing of the school students they will teach in their future careers. HPE teachers are in a unique position of being responsible for the development of knowledge, skills, and attitudes related to health, physical activity, and nutrition in children and adolescents through compulsory curriculum requirements and elective school programs. They therefore have the potential to influence children’s behaviour through direct instruction and role-modelling health behaviours. Teachers are well recognised as significant role models for their students [14]. Several early studies have shown that role modelling plays a significant role in the success of HPE programs and that there is a strong potential for positive role modelling when students observe their teachers engaging in sensible and healthy lifestyle behaviours [19–21]. However, very little research has investigated the modelling of behaviours that may cause harm. Although not empirically tested, the known potential for positive role modelling indicates that there is the potential for body-related attitudes, disordered eating, and exercise behaviours to be transferred from HPE teachers to students [3, 22]. This is a particular concern in relation to the development of eating disorders.

The aim of this study was to compare the body image, eating, and exercise attitudes and behaviours of first-year education university students who were specialising in HPE, with those who were not specialising in this area. Previous research on this topic used male and female students from a range of year levels at university [4] or excluded males [6]. The present study is unique in that it includes both male and female students, and focuses only on students in their first year of a teacher education course. It is not currently whether students with higher levels of eating and exercise concerns are attracted to a HPE degree, or whether students enrolled in HPE degrees might develop disordered eating attitudes and behaviours over the course of the degree. Thus, in focusing only on first year students, this study aims to identify whether HPE students enter their degree with higher levels of body dissatisfaction, disordered eating and excessive exercise than other education students. This will lay the foundation for future research to understand whether we attract or create HPE teachers with disordered eating attitudes and behaviours.

## Method

### Participants

Participants were 596 first-year university students from three universities in Australia: La Trobe University, Victoria University and the University of Western Sydney. Students were enrolled in an undergraduate education degree, and were studying to become either HPE teachers (*n* = 249), or non-HPE teachers (*n* = 347). Non-HPE teachers were studying to become primary school teachers or secondary school teachers specialising in Mathematics, English, and other teaching areas. Data were collected from three universities, in two states of Australia, across 4 years from 2012 to 2015. There were two cohorts from each university. Data from participants for whom gender was unknown were omitted from analyses. One participant identified their gender as “other” (0.2%) and six participants did not provide a response (1.0%). Thus, the final sample size for analyses was *N* = 589 (HPE: *n* = 246; non-HPE: *n* = 343). Participants’ ages ranged from 17 to 58 years (*M*_*age*_ = 21.10, *SD* = 5.1). Information on age was not provided by 12 participants. The majority of participants were women (*n* = 354, 60.1%; male *n* = 235, 39.9%), which reflects the typical gender balance of undergraduate education courses in Australia [23].

### Measures

#### Demographics

Participants provided self-report data indicating the university at which they were studying and the degree they were studying. Participants also indicated their ethnicity, age, height, and weight.

#### Body image

Three measures of body image were used. The body dissatisfaction subscale of the Eating Disorders Inventory-2 (EDI-2) [24] assessed body dissatisfaction. This subscale usually includes nine items asking about levels of satisfaction/dissatisfaction with stomach, hips, thighs, and buttocks where there is one positive and one negatively worded question for each and one global satisfaction item. The La Trobe University Human Ethics Committee requested that the negatively worded items (e.g., “I think that my stomach is too big”) were removed from the questionnaire to reduce duplication and a negative focus of the questionnaire. Thus, for the first La Trobe University cohort, only the four positively worded items for body dissatisfaction (e.g., “I think my stomach is just the right size”) and the global item “I feel satisfied with the shape of my body” were included. In subsequent cohorts, modifications to the ethics approval and questionnaire were made, and all nine items were included in the questionnaire. However, the analyses presented here include only the five items of this scale that are common to all participant cohorts. In the cohorts for which all items were available, correlations between total scores which included all nine items and scores which included five items were calculated. The strength of the correlation for men, *r*(155) = .79, *p* < .001, was significantly lower, *z* = -6.25, *p* < .00, than the correlation for women, *r*(212) = .94, *p* < .001, suggesting that the comparability of the nine and five item versions is less for men than women. However, the correlations were high for both genders, indicating the similarity of the 5-item version to the original 9-item scale and supporting the use of the abbreviated version.

The seven items of the drive for thinness subscale of the EDI-2 [24] were included to determine drive for thinness (e.g., “I am preoccupied with the desire to be thinner”). Participant responses to items for both the body dissatisfaction and drive for thinness subscales were recorded on a 6-point scale from 1 (*never*) to 6 (*always*). Item responses were reverse coded where appropriate and summed to form a total score (range: 5 to 30 for body dissatisfaction; 7 to 42 for drive for thinness). Higher scores reflect higher body dissatisfaction and higher drive for thinness. Scores on both the body dissatisfaction and drive for thinness subscales have been shown to be invariant across gender in early adult samples, suggesting the scales are comparable for use with both men and women [25]. Construct validity of both subscales has also been supported [25]. Internal validity of scores has been shown to be adequate in adult samples [26]. Cronbach’s alpha in the current study was .84 and .86 for body dissatisfaction, and .93 and .88 for drive for thinness, for men and women, respectively.

Drive for muscularity was assessed with the 15-item Drive for Muscularity Scale [27]. Responses to items such as “I wish I were more muscular” are recorded on a 6-point scale from 1 (*always*) to 6 (*never*). Item responses are reverse coded prior to summing for a total score (range: 15 to 90) so that higher scores reflect higher drive for muscularity (note that a total scale score can also be calculated from the mean of item responses). Supporting construct validity, previous factor analyses indicate a one-factor scale for use with both men and women [28]. Construct validity is further supported in men and women with scores on the Drive for Muscularity Scale having been shown to be significantly correlated with muscularity-related body comparison and with social physique anxiety [29]. Scale scores have shown adequate test-retest reliability and internal consistency in men [30]. Cronbach’s alpha in the current study was .91 and .90 for males and females, respectively.

#### Excessive exercise

Various definitions for excessive, pathological, or obligatory exercise is exist, but generally involve the amount of exercise as well as the level of commitment to it and implications for work and social life [31]. In this study, level of excessive exercise was assessed with the Obligatory Exercise Questionnaire [5]. The 20-item questionnaire assesses quantitative and qualitative aspects of compulsion, or obligation to exercise. Responses to items such as “When I don’t exercise I feel guilty” are recorded on a 4-point scale from 1 (*always*) to 4 (*often*). Total scores are calculated from the sum of item responses (range: 20–80) and higher scores reflect higher levels of obligation to exercise. A cut-off score of ≥ 50 can also be used to classify participants as “obligatory exercisers” [5]. In samples of adult males and females, scale scores have been found to have higher correlations with measures of compulsive exercise than with measures of exercise addiction, supporting construct validity [31]. Cronbach’s alpha in the current study was .90 for both male and female samples.

#### Weight change behaviours

Engagement in dieting, disordered eating, and moderate and unhealthy weight change behaviours was assessed with 17 items used in prior research to examine weight change behaviours [4]. Sample items are use of a “diet from a magazine or book,” “fasting,” “vomiting,” use of “anabolic steroids,” and excessive exercise (more than 2 h per day not related to your professional career or training). Participants were initially asked whether they had tried to change their weight in the past 12 months. Those who responded positively were then asked to indicate how frequently they had used each method, with response options being 1 (*in the last month*), 2 (*in the last 12 months*), or 3 (*never*). Responses were recoded to dichotomous format so that zero indicated that the behaviour had not been used and one indicated that the behaviour had been used in the past 12 months, or the past month. Items were analysed separately, so scale score characteristics were not calculated.

### Procedure

Approval for the study was received from the La Trobe University, Victoria University, and University of Western Sydney Human Ethics Committees. The data presented in this paper were collected as a part of a larger longitudinal study that intended to compare students at the beginning and end of their tertiary education. For this first data collection time-point, recruitment and survey completion was conducted during the first 3 weeks of classes starting, in order to capture students’ attitudes and behaviours at the point of initiating study at university. To do this, arrangements were made with the coordinators of large first-year units of study to enable data collection or recruitment to occur through their classes. Researchers visited the lectures of students in order to provide information about the study, invite survey participation, and conduct hard-copy data collection during class time, supervised by the researchers, for the first cohort at all universities. The second University of Western Sydney cohort was also recruited and conducted in this manner but the questionnaires were completed online. For the second La Trobe University and Victoria University cohorts, researchers visited lectures to introduce and provide information about the study, and then emailed the students the link to the survey. For these cohorts, survey completion was conducted online, was not supervised, and resulted in much lower participant numbers. Exact numbers of students present in class or emailed were not recorded, so response rates are not calculated. All participants provided written informed consent to participate either online through Qualtrics or by signing a consent form.

### Data analysis

Independent-samples *t*-tests compared age and BMI for HPE and non-HPE groups. Analyses of covariance controlling for demographic variables that differed between the two participant groups examined differences between HPE and non-HPE participants for body dissatisfaction, drive for thinness, and drive for muscularity. A series of multiple logistic regression analyses was conducted separately for men and women to examine associations between degree (HPE and non-HPE) and the dichotomous dependent variables for each of the weight change behaviours, controlling for demographic variables that differed between the two participant groups.

## Results

### Participant characteristics

Height and weight information was provided by 192 men and 241 women (81.7% and 68.1% of participants, respectively), from which BMI was calculated. Participants’ BMI ranged from 17.0 to 59.5. Mean BMI for both men and women was in the upper end of the normal weight range (*M*_*men*_ = 24.93, *SD* = 4.26; *M*_*women*_ = 24.57, *SD* = 5.63). A small proportion of participants had a BMI < 18.5 (men 1.6%; women 5.0%), the majority of the samples were between 18.5 and 24.9 (men 55.2%; women 59.3%), a moderate proportion was between 25.0 and 29.9 (men 33.9%; women 21.6%), and small proportions were between 30.0 and 34.9 (men 6.8%; women 10.0%), 35.0 and 39.9 (men 1.6%; women 2.9%), and greater than or equal to 40 (men 1.0%; women 1.2%).

Results from independent-samples *t*-tests showed that male and female HPE participants (*M*_*men*_ = 20.52, *SD* = 3.00; *M*_*women*_ = 19.77, *SD* = 1.99) were significantly younger than non-HPE participants (*M*_*men*_ = 21.74, *SD* = 5.13; *M*_*women*_ = 21.79, *SD* = 6.80), *t*(227) = 2.13, *p* = .035, *ƞ* ^2^ = .020 and *t*(347) = 4.18, *p* < .001, *ƞ* ^2^ = .048 for men and women respectively. In addition, female HPE participants (*M* = 22.98, *SD* = 3.46) had significantly lower BMI than female non-HPE participants (*M* = 25.42, *SD* = 6.35), *t*(239) = 3.87, *p* < .001, *ƞ* ^2^ = .059. The difference between male HPE (*M* = 24.75, *SD* = 3.72) and non-HPE participants (*M* = 25.16, *SD* = 4.89) for BMI was not significant *t*(190) = 0.67, *p* = .504, *ƞ* ^2^ = .002.

### Body image and excessive exercise

Means and standard deviations for body dissatisfaction, drive for thinness, drive for muscularity, and excessive exercise are shown in Table 1. To examine differences between participant groups on these variables, analyses of covariance (ANCOVA) were conducted separately for men and women with age as the covariate. These revealed that male HPE participants had significantly higher drive for muscularity than male non-HPE participants did. The effect size was small (see Table 1). Scores for the Obligatory Exercise Questionnaire were significantly higher for both male and female HPE participants than their non-HPE counterparts. Effect sizes were small and medium for men and women, respectively. There were no other significant differences between HPE and non-HPE participants for either men or women. Analyses were repeated with BMI also included as a covariate for the smaller subsample of women with BMI data available, and outcomes did not change.

**Table 1:** Summary statistics for analyses of covariance controlling for age for body dissatisfaction, drive for thinness, and drive for muscularity for male and female HPE and non-HPE participants

	Men								Women							
	HPE^a^		Non-HPE^b^				All men		HPE^c^		Non-HPE^d^				All women	
	Mean	*SD*	Mean	*SD*	*F*	*η*_*p*_^2^	Mean	*SD*	Mean	*SD*	Mean	*SD*	*F*	*η*_*p*_^2^	Mean	*SD*
Body dissatisfaction	16.81	5.70	17.62	6.80	0.84	0.004	17.17	6.17	18.47	5.76	18.39	6.65	0.09	<0.001	18.31	6.38
Drive for thinness	23.25	10.63	23.21	11.75	<0.001	<0.001	23.41	11.12	24.42	8.76	23.85	8.77	0.001	<0.001	24.03	8.71
Drive for muscularity	47.13	15.87	41.56	14.74	5.70*^	0.026	44.87	15.85	28.50	12.11	25.90	10.73	3.17^¶^	0.010	26.77	11.21
Obligatory exercise	50.66	10.33	45.46	11.17	10.71**	0.047	48.45	11.09	48.18	11.30	41.66	9.95	24.98***	0.072	43.86	10.82
	*n*	%	*n*	%	*z*		*n*	%	*n*	%	*n*	%	*z*		*n*	%
Percentage of obligatory exercisers	64	51.2	28	28.6	3.40***		92	41.3	44	39.3	42	19.8	3.78***		86	26.5

* *p* < .05; **p < .01, ***p < .001, ^¶^ *p* < 1.0

*Note:* Sample sizes differed due to missing data. The following participant numbers for each cohort are provided as a guide: ^a^ *n* = 116–123; ^b^ *n* = 92–100; ^c^ *n* = 107–109; ^d^ *n* = 207–219; ^ Following Bonferroni adjustment, this result was no longer significant at an alpha of *p* < .01

The proportion of HPE compared with non-HPE participants identified as obligatory exercisers were compared with the two-proportion *z*-test. These revealed that significantly greater proportions of both male and female HPE than non-HPE participants were classified as obligatory exercisers (see Table 1).

### Methods of weight loss

The frequencies of use of specific dieting, weight change, and disordered eating behaviours are presented in Table 2 according to gender and degree. Of those engaged in weight change behaviours, a little under half of the male undergraduates (40%) indicated that they were on a diet that they created themselves, and 41% were consuming protein products. Among the females, over half of participants (57%) indicated that they were on a diet that they created themselves, and 26% had consumed protein shakes or powders. Large proportions of men and women indicated that they used exercise to change their weight (67% men, 81% women), and around half were drinking water to change their weight and shape (46% men, 58% women).

**Table 2:** Frequency of dieting, weight change, and disordered eating behaviours for male and female HPE and non-HPE participants

	Males						Females					
	HPE^a^		Non-HPE^b^		All men		HPE^c^		Non-HPE^d^		All women	
	*n*	%	*n*	%	*n*	%	*n*	%	*n*	%	*n*	%
Dieting and weight change behaviours												
Not eating between meals	27	21.6	21	20.0	48	20.9	48	42.5	115	49.6	163	47.2
Drinking water	57	47.2	48	45.3	107	46.3	72	61.5	133	56.6	205	58.2
Diet created by self	58	46.4	33	31.7	91	39.7	67	59.3	128	55.2	195	56.5
Diet from magazine	10	8.1	7	6.6	17	7.4	18	15.7	38	16.5	56	16.2
Slimming tea	8	6.5	1	0.9	9	3.9	15	12.9	26	11.2	41	11.7
Skipping meals	18	14.4	21	19.8	39	16.9	39	33.9	87	37.3	126	36.2
Exercise	88	69.8	67	64.4	155	67.4	90	78.9	193	82.1	283	81.1
Detox products	6	4.8	6	5.7	12	5.2	12	10.5	23	9.8	35	10.0
Protein shakes/snacks/powders	58	46.0	37	35.6	95	41.3	33	28.7	58	24.8	91	26.1
Creatine	23	18.5	18	17.0	41	17.8	5	4.3	3	1.3	8	2.3
Disordered eating and unhealthy weight change behaviours												
Smoking	9	7.3	5	4.7	14	6.1	4	3.5	20	8.6	24	6.9
Fasting	12	9.7	7	6.6	19	8.3	21	18.3	38	16.3	59	17.0
Laxatives	6	4.8	1	0.9	7	3.0	5	4.4	7	3.0	12	3.5
Vomiting	9	7.3	4	3.8	13	5.7	3	2.6	11	4.7	14	4.0
Slimming pills	7	5.6	8	7.5	15	6.5	7	6.1	12	5.2	19	5.4
Excessive exercise	48	38.4	30	28.6	78	33.9	43	37.4	46	19.8	89	25.6
Anabolic steroids	9	7.3	1	1.0	10	4.4	1	0.9	0	0.0	1	0.3

*Note:* Sample sizes differed due to missing data, but percentages were calculated with exact numbers for each variable. The following participant numbers for each cohort are provided as a guide: ^a^ *n* = 123–126; ^b^ *n* = 104–106; ^c^ *n* = 113–117; ^d^ *n* = 231–235

Results from logistic regression analyses, controlling for age, presented in Table 3, showed that male HPE participants were 2.0 times more likely than male non-HPE participants to follow a diet created by themselves. In relation to disordered eating and unhealthy weight control behaviours, male HPE participants were 14.3 times more likely to take anabolic steroids than were non-HPE participants. Age was also a significant predictor of dieting, weight change, and disordered eating behaviours, with older participants more likely to not eat between meals, engage in diets they created themselves, use detox products, fast for weight change purposes, and use anabolic steroids than younger participants. Many of the significant differences between the cohorts did not survive Bonferroni correction for Type 1 error.

**Table 3:** Summary statistics for logistic regression analyses examining dieting, weight change, and disordered eating behaviours for men

Dependent variable	Predictor variable	*β*	SE *β*	OR	95% CI	*p*
Dieting and weight change behaviours						
Not eating between meals	Age	0.13	0.04	1.14	1.05, 1.25	**.002**
	Degree^a^	−0.33	0.35	0.72	0.37, 1.42	.346
Drinking water	Age	0.05	0.04	1.05	0.98, 1.12	.208
	Degree^a^	−0.66	0.27	0.94	0.55, 1.60	.808
Diet created by self	Age	0.14	0.05	1.15	1.05, 1.25	**.002**
	Degree^a^	−0.71	0.29	0.49	0.28, 0.87	**.015**
Diet from magazine	Age	0.06	0.04	1.07	0.98, 1.16	.151
	Degree^a^	−0.21	0.54	0.81	0.28, 2.33	.697
Slimming tea	Age	0.10	0.06	1.10	0.98, 1.24	.100
	Degree^a^	−2.32	1.19	0.10	0.01, 1.02	.052
Skipping meals	Age	0.07	0.04	1.07	1.00, 1.16	.068
	Degree^a^	0.18	0.37	1.20	0.58, 2.47	.619
Exercise	Age	0.09	0.05	1.10	1.00, 1.21	.062
	Degree^a^	−0.29	0.29	0.75	0.42, 1.32	.318
Detox products	Age	0.10	0.05	1.11	1.01, 1.21	**.031**
	Degree^a^	−0.05	0.62	0.95	0.28, 3.22	.932
Protein shakes / snacks / powders	Age	0.04	0.04	1.05	0.98, 1.12	.203
	Degree^a^	−0.44	0.28	0.65	0.37, 1.12	.118
Creatine	Age	0.02	0.04	1.02	0.94, 1.10	.639
	Degree^a^	−0.07	0.36	0.94	0.46, 1.91	.856
Disordered eating and unhealthy weight change behaviours						
Smoking	Age	0.06	0.05	1.06	0.97, 1.17	.181
	Degree^a^	−0.57	0.60	0.57	0.18, 1.83	.342
Fasting	Age	0.11	0.05	1.12	1.02, 1.23	**.014**
	Degree^a^	−0.66	0.53	0.52	0.18, 1.46	.212
Laxatives	Age	0.07	0.07	1.07	0.93, 1.23	.353
	Degree^a^	−1.83	1.14	0.16	0.02, 1.52	.111
Vomiting	Age	<0.01	0.08	1.00	0.86, 1.16	.982
	Degree^a^	−0.67	0.63	0.51	0.15, 1.74	.283
Slimming pills	Age	0.04	0.05	1.04	0.94, 1.14	.480
	Degree^a^	0.28	0.55	1.32	0.45, 3.85	.610
Excessive exercise	Age	0.02	0.03	1.02	0.95, 1.09	.536
	Degree^a^	−0.42	0.29	0.66	0.37 1.16	.148
Anabolic steroids	Age	0.12	0.05	1.12	1.01, 1.25	**.032**
	Degree^a^	−2.63	1.27	0.07	0.06, 0.86	**.037**

*Note:* ^a^ Course HPE = 0, non-HPE = 1; Bonferroni adjusted alpha was *p* < .003; Significant results are bolded

Almost one third of female participants did not provide height or weight information; thus, BMI could not be calculated for the full sample. Consequently, logistic regression analyses for women were initially conducted controlling for age only, and were repeated controlling for both age and BMI for the subsample for which BMI was available. Analyses controlling for age revealed that HPE females were 2.1 times more likely than non-HPE participants to engage in excessive exercise to change their weight. Age was not a significant predictor of weight change behaviours for women. Summary statistics for logistic regression analyses for women are shown in Table 4. Note that logistic regression analyses could not be conducted for use of anabolic steroids as only one woman across both participant samples endorsed using anabolic steroids.

**Table 4:** Summary statistics for logistic regression analyses examining dieting, weight change, and disordered eating behaviours for women

Dependent variable	Predictor variable	*β*	SE β	OR	95% CI	*p*
Dieting and weight change behaviours						
Not eating between meals	Age	−0.02	0.02	0.99	0.95, 1.02	.418
	Degree^a^	0.33	0.24	1.39	0.87, 2.20	.169
Drinking water	Age	−0.04	0.02	0.97	0.93, 1.00	.074
	Degree^a^	−0.09	0.24	0.92	0.58, 1.46	.707
Diet created by self	Age	−0.02	0.02	0.98	0.94, 1.02	.250
	Degree^a^	−0.11	0.24	0.89	0.56, 1.43	.637
Diet from magazine	Age	<0.01	0.03	1.00	0.96, 1.05	.864
	Degree^a^	0.17	0.33	1.18	0.62, 2.25	.608
Slimming tea	Age	−0.02	0.04	0.98	0.92, 1.05	.560
	Degree^a^	−0.15	0.35	0.86	0.43, 1.71	.665
Skipping meals	Age	−0.01	0.02	0.99	0.95, 1.03	.645
	Degree^a^	0.22	0.25	1.25	0.77, 2.02	.367
Exercise	Age	−0.02	0.02	0.98	0.94, 1.02	.288
	Degree^a^	0.31	0.29	1.37	0.77, 2.43	.290
Detox products	Age	−0.01	0.04	0.99	0.92, 1.06	.743
	Degree^a^	0.02	0.39	1.02	0.47, 2.19	.965
Protein shakes / snacks / powders	Age	−0.01	0.02	0.99	0.95, 1.04	.803
	Degree^a^	−0.13	0.26	0.88	0.53, 1.48	.630
Creatine	Age	0.07	0.05	1.07	0.97, 1.17	.169
	Degree^a^	−1.54	0.82	0.22	0.43, 1.07	.061
Disordered eating and unhealthy weight change behaviours						
Smoking	Age	0.01	0.03	1.01	0.95, 1.08	.683
	Degree^a^	0.92	0.57	2.50	0.83, 7.58	.105
Fasting	Age	−0.03	0.03	0.97	0.91, 1.03	.301
	Degree^a^	−0.10	0.30	0.90	0.50, 1.63	.732
Laxatives	Age	0.02	0.05	1.02	0.93, 1.12	.644
	Degree^a^	−0.46	0.61	0.63	0.19, 2.10	.453
Vomiting	Age	−0.05	0.07	0.48	0.82, 1.10	.475
	Degree^a^	0.67	0.66	0.31	0.53, 7.18	.314
Slimming pills	Age	0.03	0.03	1.03	0.97, 1.10	.324
	Degree^a^	−0.29	0.51	0.75	0.28, 2.02	.571
Excessive exercise	Age	−0.05	0.03	0.95	0.89, 1.02	.139
	Degree^a^	−0.76	0.26	0.47	0.28, 0.78	**.004**

*Note:* ^a^ Course HPE = 0, non-HPE = 1; Bonferroni adjusted alpha was *p* < .003; Significant results are bolded; Logistic regression analyses could not be conducted for anabolic steroids because only one participant endorsed that method of weight change

Results from analyses controlling for both age and BMI for the smaller subsample of participants for whom BMI could be calculated were somewhat different from analyses that controlled only for age. Degree remained a significant predictor of excessive exercise with HPE participants 2.6 times more likely than non-HPE participants to engage in excessive exercise. Age became a significant predictor of both drinking water to change weight and following a diet created by the self, with younger participants more likely to use these practices than older participants. In addition, BMI predicted use of skipping meals; following a diet created by both the self and a magazine; exercise; use of protein shakes, snacks, or powders; and excessive exercise, with participants with higher BMI more likely to use those weight loss methods than participants with lower BMI. However, many of the significant differences between the cohorts did not survive Bonferroni correction for Type 1 error. Summary statistics for logistic regression analyses for women controlling for both age and BMI are available in the Additional file 1: Table S1.

## Discussion

This study compared the body image and weight change behaviours of undergraduate students studying to become teachers in HPE or non-HPE fields. These findings demonstrate high levels of body dissatisfaction, excessive exercise, and drive for thinness and muscularity for both HPE and non-HPE education students. It is concerning that, at the point of starting their higher education and at the mean age of 21 years, a large proportion of male and female undergraduates were engaged in a range of behaviours to change their weight and shape.

Our hypotheses were partially supported. Overall, there were very few significant differences between the female HPE and non-HPE groups. The HPE women were significantly more likely to report engaging in excessive exercise and to score significantly higher for obligatory exercise, but there were no differences in body dissatisfaction or drive for muscularity between the female groups. There were a greater number of significant differences between HPE and non-HPE males. HPE males were significantly more likely than non-HPE males to have higher drive for muscularity and higher scores for obligatory exercise. They were also significantly more likely to report being on a diet they had created themselves, and to use anabolic steroids. The percentage of HPE students using anabolic steroids in the past 12 months (7.3%) was particularly noteworthy.

The reporting of anabolic steroid use in this study may need to be interpreted with caution as previous research has suggested that estimates of anabolic steroid use are sensitive to the wording of the questions used, and some assessment questions can lead to false-positive responses due to lack of differentiation between anabolic steroids and both generic steroids and other performance enhancing or body change substances [32]. The converse outcome, under-reporting of anabolic steroid use, may occur as a result of concerns about stigmatising reactions toward steroid use [33]. The method used in the current study was not likely to produce false-positive responses as the wording in the questionnaire specified *anabolic* steroid use, not simply steroid use. In addition, the item was included among other items assessing creatine and protein powder/shake use. Thus, participants were unlikely to confuse anabolic steroids with generic steroids, such as prescription corticosteroids, or with other supposed muscle building substances including creatine and protein products. It is unclear whether participants underreported steroid use, but the usage rates were comparable with previous reports for males [34], suggesting that under-reporting did not occur. Further, in terms of proportions, male HPE students reported consistent use, even relative to female groups, of vomiting, excessive exercise, protein powders, creatine, laxatives, and anabolic steroids. These findings indicate that males entering HPE degrees are engaging in behaviours that are potentially damaging to their health and wellbeing.

Our previous research reported a greater number of significant differences between HPE and non-HPE females [35]. However, the data for that study were collected at a range of time points, so some students were in their first year of study, while others were in their second, third, or fourth year of study. O’Brien and Hunter [6] found that female undergraduates studying PE who had completed nearly 3 years of their course had significantly higher rates of dieting and disordered eating behaviours than first-year PE females and first- and third-year psychology students. Longitudinal research conducted among college students in Canada (not limited to those in food- and exercise-related degrees) indicated that in general, both male and female university students experienced increases in BMI and dietary restraint, whereas depression and Eating Disorder Inventory scores remained relatively stable over the 4-year period [36]. As such, we still do not know whether students might be attracted to HPE teaching due to their own personal attitudes and preoccupation with diet and exercise [17, 37], or whether being immersed in a culture of fitness and health, surrounded by people who are similarly involved in exercise and weight control, may also increase disordered eating and exercise behaviours through social norms and reinforcement.

Differences in the findings between previous work and the present study could also be due to a shift in attitudes of female undergraduates over time from earlier studies when data were collected in the 1980s and early 2000s [35] and 2012–2015 when the data from the current study were collected. It may be that all undergraduates now have high levels of body dissatisfaction, disordered eating, and weight change behaviours, not just those in degrees related to food and exercise [38, 39]. Our research found that the proportion of men and women who reported vomiting and laxative use was double the prevalence rates reported in a recent study of Italian university students [39]. This would be consistent with recent research which has shown an increase in the prevalence of dieting and disordered eating behaviours, including steroid use, across different sectors of the Australian community [40, 41].

Interestingly, the current study also found no significant difference between HPE and non-HPE males or females on the body dissatisfaction subscale of the EDI-2. These findings are consistent with previous research. For example, our earlier work found no difference in discrepancy scores on the Stunkard Figure Rating Scale for male or female participants [35]. In addition, O’Brien and Hunter [6] found no differences on the weight concern subscale of the Body Esteem Scale between female HPE and female psychology students. It is interesting to note that in these previous studies, although HPE participants did not score higher than non-HPE participants in terms of their body dissatisfaction, they were more likely to participate in weight change and disordered eating behaviours. Some researchers suggest that the lower levels of body dissatisfaction among female PE students may be present because the (sometimes extreme) eating and exercise behaviours that the female students are engaging in are successful in reducing their weight to a socially acceptable level [6]. This may also be the case with the present findings, as the HPE females had both significantly lower BMI and significantly higher excessive exercise levels than their non-HPE counterparts.

Investigation of the personal eating and exercise behaviours of teachers is crucial as they play a significant role in providing health education and managing the school environment. HPE teachers in particular are charged with a critical role in fostering positive attitudes towards health and movement and they teach directly about nutrition and physical activity. If these teachers are experiencing high levels of personal eating and exercise behaviours, there is the potential that these could be unknowingly transferred to students through direct and indirect means [3]. Previous research indicates the strong impact of teacher role modelling, especially for health behaviours [14, 19, 42]. The findings of this study—that HPE men in particular enter their degrees with significantly higher levels of dieting, drive for muscularity, obligatory exercise, and steroid use—provides a strong rationale for the inclusion of interventions to address body dissatisfaction and disordered eating and exercise behaviours during the preparation of these professionals for their teaching career [43]. This might improve future teachers’ capacity to encourage positive body image, nutrition, and physical activity without modelling inappropriate attitudes and behaviours.

Although the aim of this study was to compare students in HPE and non-HPE specialisations in teacher education programs, our study also contributes relatively unique prevalence data in terms of the use of weight change behaviours among a large cohort of Australians. Results of the logistic regression analyses indicated that the effect of age was significant for males in terms of the likelihood of participants reporting that they did not eat between meals, were on a diet they had created, used detox products, took steroids, and engaged in fasting, such that older men were significantly more likely to be engaging in these behaviours. Among the smaller proportion of women who had reported their height and weight, the effect of age was significant for creating their own diet and drinking water between meals, and younger women were more likely to be engaging in these behaviours. This data suggests that it is important for researchers to control for age in analyses.

The findings of this study need to be considered in the context of several limitations. First, there were some variations in the data collection procedure in that some students completed the questionnaires in class in a relatively controlled environment, and others completed them online at a time that suited them. Valid measurements of body image and body dissatisfaction and disordered eating behaviours are difficult among men as they are generally motivated by muscularity rather than thinness [44]. Some of the items of the questionnaire differed slightly in terms of how they were presented in the online and paper-based versions. In addition, the request to remove some questionnaire items by the ethics committee at one of the participating universities meant that we did not ask all of the questions that would normally have been included in that standardised scale. The potential for Type 1 error means that many of the findings should be interpreted with caution.

## Conclusions

This study found that males who are in their first year of preparing to become HPE teachers are significantly more likely to have higher levels of drive for muscularity and excessive exercise and to be engaging in a range of dieting and disordered eating behaviours than other male teacher education students. The only significant difference between female HPE and non-HPE students was the levels of excessive and obligatory exercise. These findings suggest that further investigation of male undergraduates studying HPE is needed, and that teacher education programs may need to incorporate materials that focus on both the personal and professional education of future graduates. This will ensure their own health and wellbeing, and that of their future students.

## Additional file

Additional file 1: Table S1. Summary Statistics for Logistic Regression Analyses Examining Dieting, Weight Change, and Disordered Eating Behaviours for Females, Controlling for Age and BMI. (DOC 95 kb)

## Abbreviations

ANCOVA: Analyses of covariance
BMI: Body mass index
HPE: Health and physical education
PE: Physical education
USA: United States of America
