# Supplementary material for: Body dissatisfaction, excessive exercise, and weight change strategies used by first-year undergraduate students: comparing health and physical education and other education students
Source: J Eat Disord. 2017 Apr 3;5:10. doi: 10.1186/s40337-016-0133-z (PMC5376693; doi:10.1186/s40337-016-0133-z)
Supplement: Additional file 1: Table S1. — Summary Statistics for Logistic Regression Analyses Examining Dieting, Weight Change, and Disordered Eating Behaviours for Females, Controlling for Age and BMI. (DOC 95 kb) [file 40337_2016_133_MOESM1_ESM.doc]

##### Additional file 1: Table S1

*Summary Statistics for Logistic Regression Analyses Examining Dieting, Weight Change, and Disordered Eating Behaviours for Females, Controlling for Age and BMI*

| Dependent variable | Predictor variable | *β* | SE *β* | OR | 95% CI | *p* |
| --- | --- | --- | --- | --- | --- | --- |
| Dieting and weight change behaviours | |  |  |  |  |  |
| Not eating between meals | Age | -0.02 | 0.022 | 0.983 | 0.94, 1.03 | .445 |
|  | BMI | 0.03 | 0.03 | 1.03 | 0.98, 1.08 | .290 |
|  | Degree^a^ | 0.40 | 0.30 | 1.49 | 0.84, 2.45 | .174 |
| Drinking water | Age | -0.05 | 0.02 | 0.95 | 0.91, 0.10 | **.038** |
|  | BMI | 0.04 | 0.03 | 1.04 | 0.99, 1.10 | .116 |
|  | Degree^a^ | 0.02 | 0.29 | 1.02 | 0.58, 1.81 | .938 |
| Diet created by self | Age | -0.05 | 0.02 | 0.948 | 0.91, 0.99 | **.027** |
|  | BMI | 0.06 | 0.03 | 1.06 | 1.01, 1.13 | **.030** |
|  | Degree^a^ | 0.08 | 0.29 | 1.09 | 0.61, 1.94 | .776 |
| Diet from magazine | Age | -0.03 | 0.03 | 0.97 | 0.91, 1.04 | .340 |
|  | BMI | 0.05 | 0.03 | 1.05 | 1.00, 1.12 | **.039** |
|  | Degree^a^ | -0.01 | 0.38 | 0.99 | 0.47, 2.07 | .975 |
| Slimming tea | Age | -0.04 | 0.05 | 0.96 | 0.88, 1.06 | .337 |
|  | BMI | 0.02 | 0.03 | 1.02 | 0.95, 1.08 | .611 |
|  | Degree^a^ | -0.39 | 0.39 | 0.68 | 0.32, 1.45 | .318 |
| Skipping meals | Age | -0.04 | 0.03 | 0.96 | 0.91, 1.02 | .131 |
|  | BMI | 0.05 | 0.03 | 1.05 | 1.00, 1.11 | .**047** |
|  | Degree^a^ | 0.50 | 0.31 | 1.65 | 0.90, 3.05 | .106 |
| Exercise | Age | -0.04 | 0.03 | 0.96 | 0.91, 1.02 | .192 |
|  | BMI | 0.10 | 0.05 | 1.03 | 1.01, 1.22 | **.032** |
|  | Degree^a^ | 0.48 | 0.37 | 1.62 | 0.78, 3.45 | .194 |
| Detox products | Age | -0.04 | 0.05 | 0.96 | 0.88, 1.05 | .408 |
|  | BMI | 0.03 | 0.03 | 1.04 | 0.97, 1.10 | .288 |
|  | Degree^a^ | -0.14 | 0.44 | 0.87 | 0.37, 20.7 | .760 |
| Protein shakes / snacks / powders | Age | -0.03 | 0.03 | 0.98 | 0.92, 1.03 | .365 |
|  | BMI | 0.10 | 0.03 | 1.10 | 1.04, 1.17 | **.001** |
|  | Degree^a^ | -0.38 | 0.33 | 0.68 | 0.36, 1.29 | .240 |
| Creatine | Age | 0.05 | 0.05 | 1.05 | 0.95, 1.16 | .366 |
|  | BMI | 0.02 | 0.08 | 1.02 | 0.88, 1.18 | .795 |
|  | Degree^a^ | -1.23 | 0.87 | 0.29 | 0.05, 1.63 | .160 |
| Disordered eating and unhealthy weight change behaviours | | | | |  |  |
| Smoking | Age | -0.01 | 0.04 | 0.99 | 0.91, 1.07 | .787 |
|  | BMI | -.01 | 0.04 | 0.99 | 0.91 1.08 | .761 |
|  | Degree^a^ | 1.15 | 0.66 | 3.16 | 0.91, 11.43 | .080 |
| Fasting | Age | -0.07 | 0.05 | 0.94 | 0.85, 1.03 | .152 |
|  | BMI | 0.03 | 0.03 | 1.03 | 0.97, 1.09 | .294 |
|  | Degree^a^ | 0.11 | 0.38 | 1.12 | 0.53, 2.37 | .772 |
| Laxatives | Age | 0.03 | 0.05 | 1.03 | 0.93, 1.13 | .594 |
|  | BMI | 0.00 | 0.07 | 1.00 | 0.88, 1.14 | .981 |
|  | Degree^a^ | -0.79 | 0.70 | 0.46 | 0.12, 1.78 | .259 |
| Vomiting | Age | -0.09 | 0.12 | 0.92 | 0.73, 1.15 | .460 |
|  | BMI | 0.02 | 0.06 | 1.02 | 0.92, 1.14 | .678 |
|  | Degree^a^ | 0.08 | 0.75 | 1.09 | 0.25, 4.69 | .910 |
| Slimming pills | Age | 0.01 | 0.04 | 1.01 | 0.93, 1.10 | .820 |
|  | BMI | 0.07 | 0.04 | 1.08 | 1.00, 1.15 | **.043** |
|  | Degree^a^ | -0.64 | 0.56 | 0.53 | 0.17, 1.60 | .260 |
| Excessive exercise | Age | -0.06 | 0.04 | 0.94 | 0.87, 1.02 | .123 |
|  | BMI | 0.06 | 0.03 | 1.06 | 1.01, 1.12 | **.032** |
|  | Degree^a^ | -0.96 | 0.32 | 0.38 | 0.20, 0.72 | **.003** |

*Note:* ^a^ Course HPE = 0, non-HPE = 1.; Bonferroni adjusted alpha was p<.003; Logistic regression analyses could not be conducted for anabolic steroids because only one participant endorsed that method of weight change.
